# Supplementary material for: Programmable RNA detection with CRISPR-Cas12a
Source: Nat Commun. 2023 Sep 5;14:5409. doi: 10.1038/s41467-023-41006-1 (PMC10480431; doi:10.1038/s41467-023-41006-1)
Supplement: Supplementary file 1 — Supplementary Information [file 41467_2023_41006_MOESM1_ESM.pdf]

Table S1: List of crRNA used in this study (5'→3'). The spacer region of SAHARA crRNAs is colored to indicate the positions bound by S12 activators (blue) and the target DNA or RNA (purple):

**Name**	**Sequence**	**Figure**
crGFP-3'DNA7 (ENHANCE)	UAAUUUCUACUAAGUGUAGAUCUC AGGGCGGACUGGGUGCUTATTATT	Fig. 1b-g, Fig. 2b-h
crGFP-WT	UAAUUUCUACUAAGUGUAGAUCUC AGGGCGGACUGGGUGCU	Fig. 2f-h, Fig. 3b-h,
crGFP-SH	UAAUUUCUACUAAGUGUAGAUGA UUAGCAUUAACUCAGGGCGGAC	Fig. 3b-g, Fig. 7b-d
cr155-Head-SH	UAAUUUCUACUAAGUGUAGAUCUC AGGGCGGACGAUUAGCAUUAA	Fig. 4e,f
cr155-Tail-SH	UAAUUUCUACUAAGUGUAGAUCUC AGGGCGGACACCCCUAUCAC	Fig. 4e,f
cr155-Head-SH v2	UAAUUUCUACUAAGUGUAGAUAU GGUGAGCAAGGAUUAGCAUUAA	Fig. 7b-d
crHCV-Head-SH	UAAUUUCUACUAAGUGUAGAUCUC AGGGCGGACGUACCACAAGGC	Fig. 4b,c
crHCV-Mid-SH	UAAUUUCUACUAAGUGUAGAUCUC AGGGCGGACGAUGCACGGUCU	Fig. 4b,c
crHCV-Tail-SH	UAAUUUCUACUAAGUGUAGAUCUC AGGGCGGACGAGGUUUAGGAU	Fig. 4b,c, Fig. 5c-e, Fig. 6e-g, Fig. 6h-j, Fig. 7b-d, Fig. 7f,g
crHCV-Tail-SH-25%GC	UAAUUUCUACUAAGUGUAGAUAUC AUAUCUGAAGAGGUUUAGGAU	Fig. 6e-g
crHCV-Tail-SH-33%GC	UAAUUUCUACUAAGUGUAGAUAUC AGAUCUGAAGAGGUUUAGGAU	Fig. 6e-g
crHCV-Tail-SH-50%GC	UAAUUUCUACUAAGUGUAGAUCUC AGAUCGGAAGAGGUUUAGGAU	Fig. 6e-g

Table S2: List of Target Activators used in this study (5'→3'). The mutated positions for single-point mutants of HCV are indicated by red:

Name	Sequence	Figure
GFP-20-nt	AGCACCCAGTCCGCCCTGAG	Fig. 1b-d
GFP-20-nt RNA	AGCACCCAGUCCGCCCUGAG	Fig. 3b-g, Fig. 7b-d
GFP-Pp-6-nt	CCTGAG	Fig. 1b-g
GFP-Pp-8-nt	GCCCTGAG	Fig. 1b-g
GFP-Pp-10-nt	CCGCCCTGAG	Fig. 1b-g, Fig. 2b-d
GFP-Pp-12-nt	GTCCGCCCTGAG	Fig. 1b-g
GFP-Pp-14-nt	CAGTCCGCCCTGAG	Fig. 1b-g
GFP-Pp-16-nt	CCCAGTCCGCCCTGAG	Fig. 1b-d
GFP-Pp-18-nt	CACCCAGTCCGCCCTGAG	Fig. 1b-d
GFP-Pd-6-nt	AGCACC	Fig. 1b-g
GFP-Pd-8-nt	AGCACCCA	Fig. 1b-g
GFP-Pd-10-nt	AGCACCCAGT	Fig. 1b-g, Fig. 2b-d, Fig. 2f-h
GFP-Pd-12-nt	AGCACCCAGTCC	Fig. 1b-g
GFP-Pd-14-nt	AGCACCCAGTCCGC	Fig. 1b-g
GFP-Pd-16-nt	AGCACCCAGTCCGCCC	Fig. 1b-d
GFP-Pd-18-nt	AGCACCCAGTCCGCCCTG	Fig. 1b-d
GFP-Pp-dsDNA-10-nt	CCGCCCTGAGTAAAGCG (TS)<br>GCGAAATGAGTCCCGCC (NTS)	Fig. 2b-d, Fig. 2f-h
GFP-Pd-dsDNA-10-nt	AGCACCCAGTTAAAGCG (TS)<br>GCGAAATTGACCCACGA (NTS)	Fig. 2b-d
GFP-Pp-RNA-10-nt	CCGCCCUGAG	Fig. 2b-d,
GFP-Pd-RNA-10-nt	AGCACCCAGU	Fig. 2b-d, Fig. 2f-h

HCV polypeptide precursor RNA	GCCUUGUGGUACUGCCUGAUAGGGUGCUU GCGAGUGCCCCGGGAGGUCUCGUAGACCG UGCAUCAUGAGCACAAAUCCUAAACCUC	Fig. 4b,c, Fig.6 b-j, Fig. 7b-d, Fig. 7f,g
miRNA-155 target RNA	UUAAUGCUAAUCGUGAUAGGGGU	Fig. 4e,f, Fig. 7 b-d
GFP-RNA 730-nt	GAGAGCCGCCACCAUGGUGAGCAAGGGCG AGGAGCUGUUCACCGGGGUGGUGCCCAUC CUGGUCGAGCUGGACGGCGACGUAAACGG CCACAAGUUCAGCGUGUCCGGCGAGGGCG AGGGCGAUGCCACCUACGGCAAGCUGACC CUGAAGUUCAUCUGCACCACCGGCAAGCU GCCCGUGCCCUGGCCCACCCUCGUGACCA CCCUGACCUACGGCGUGCAGUGCUUCAGC CGCUACCCCGACCACAUGAAGCAGCACGA CUUCUUCAAGUCCGCCAUGCCCGAAGGCU ACGUCCAGGAGCGCACCAUCUUCUUCAAG GACGACGGCAACUACAAGACCCGCGCCGA GGUGAAGUUCGAGGGCGACACCCUGGUGA ACCGCAUCGAGCUGAAGGGCAUCGACUUC AAGGAGGACGGCAACAUCCUGGGGCACAA GCUGGAGUACAACUACAACAGCCACAACG UCUAUAUCAUGGCCGACAAGCAGAAGAAC GGCAUCAAGGUGAACUUCAAGAUCCGCCA CAACAUCGAGGACGGCAGCGUGCAGCUCG CCGACCACUACCAGCAGAACACCCCCAUC GGCGACGGCCCCGUGCUGCUGCCCGACAA CCACUACCUGAGCACCCAGUCCGCCCUGA GCAAAGACCCCAACGAGAAGCGCGAUCAC AUGGUCCUGCUGGAGUUCGUGACCGCCGC CGGGAUCACUCUCGGCAUGGACGAGCUGU ACAAG	Fig. 3b-d
HCV full-length WT **(3' -> 5')**	GAGTCCCGCCTGCTCCAAATCCTA	Fig. 5c-e
HCV full-length M13 **(3' -> 5')**	GAGTCCCGCCTG**G**TCCAAATCCTA	Fig. 5c-e
HCV full-length M14 **(3' -> 5')**	GAGTCCCGCCTGC**G**CCAAATCCTA	Fig. 5c-e
HCV full-length M15 **(3' -> 5')**	GAGTCCCGCCTGCT**G**CAAATCCTA	Fig. 5c-e
HCV full-length M16 **(3' -> 5')**	GAGTCCCGCCTGCTC**G**AAATCCTA	Fig. 5c-e
HCV full-length M17 **(3' -> 5')**	GAGTCCCGCCTGCTCC**G**AATCCTA	Fig. 5c-e

HCV full-length M18 **(3' -> 5')**	GAGTCCCGCCTGCTCCA**G**ATCCTA	Fig. 5c-e
HCV full-length M19 **(3' -> 5')**	GAGTCCCGCCTGCTCCAA**G**TCCTA	Fig. 5c-e
HCV full-length M20 **(3' -> 5')**	GAGTCCCGCCTGCTCCAAA**G**CCTA	Fig. 5c-e
HCV full-length M21 **(3' -> 5')**	GAGTCCCGCCTGCTCCAAAT**G**CTA	Fig. 5c-e
HCV full-length M22 **(3' -> 5')**	GAGTCCCGCCTGCTCCAAATC**G**TA	Fig. 5c-e
HCV full-length M23 **(3' -> 5')**	GAGTCCCGCCTGCTCCAAATCC**G**A	Fig. 5c-e
HCV full-length M24 **(3' -> 5')**	GAGTCCCGCCTGCTCCAAATCCT**G**	Fig. 5c-e
HCV SAHARA WT **(3' -> 5')**	CTCCAAATCCTA	Fig. 5c-e
HCV SAHARA M13 **(3' -> 5')**	**G**TCCAAATCCTA	Fig. 5c-e
HCV SAHARA M14 **(3' -> 5')**	C**G**CCAAATCCTA	Fig. 5c-e
HCV SAHARA M15 **(3' -> 5')**	CT**G**CAAATCCTA	Fig. 5c-e
HCV SAHARA M16 **(3' -> 5')**	CTC**G**AAATCCTA	Fig. 5c-e
HCV SAHARA M17 **(3' -> 5')**	CTCC**G**AATCCTA	Fig. 5c-e
HCV SAHARA M18 **(3' -> 5')**	CTCCA**G**ATCCTA	Fig. 5c-e
HCV SAHARA M19 **(3' -> 5')**	CTCCAA**G**TCCTA	Fig. 5c-e
HCV SAHARA M20 **(3' -> 5')**	CTCCAAA**G**CCTA	Fig. 5c-e
HCV SAHARA M21 **(3' -> 5')**	CTCCAAAT**G**CTA	Fig. 5c-e
HCV SAHARA M22 **(3' -> 5')**	CTCCAAATC**G**TA	Fig. 5c-e

HCV SAHARA M23 **(3' -> 5')**	CTCCAAATCC**G**A	Fig. 5c-e
HCV SAHARA M24 **(3' -> 5')**	CTCCAAATCCT**G**	Fig. 5c-e

Table S3: List of 'seed-region' binding S12-activators used in this study (5'→3'):

Name	Sequence	Figure
S12 for crGFP-SH	TTAATGCTAATCTAAAGCG (TS)<br>CGCTTTAGATTAGCATTAA (NTS)	Fig. 3b-g, Fig. 7b-d
S12 for crHCV-Head-SH	GTCCGCCCTGAGTAAAGCGA (TS)<br>TCGCTTTACTCAGGGCGGAC (NTS)	Fig. 4b,c
S12 for crHCV-Tail-SH	GTCCGCCCTGAGTAAAGCGA (TS)<br>TCGCTTTACTCAGGGCGGAC (NTS)	Fig. 4b,c, Fig. 6b-j, Fig. 6h-j, Fig. 7b-d, Fig. 7f,g
S12 for crHCV-Mid-SH	GTCCGCCCTGAGTAAAGCGA (TS)<br>TCGCTTTACTCAGGGCGGAC (NTS)	Fig. 4b,c
S12 for cr155-Head-SH	GTCCGCCCTGAGTAAAGCGA (TS)<br>TCGCTTTACTCAGGGCGGAC (NTS)	Fig. 4e,f
S12 for cr155-Tail-SH	GTCCGCCCTGAGTAAAGCGA (TS)<br>TCGCTTTACTCAGGGCGGAC (NTS)	Fig. 4e,f
S12 for cr155-Head v2 SH	CTTGCTCACCATTAAACAC (TS)<br>GTGTTTAATGGTGAGCAAG (NTS)	Fig. 6b-d
S12 for crHCV-Tail 25%	TTCAGATATGATTAAACAC (TS)<br>GTGTTTAATCATATCTGAA (NTS)	Fig. 6e-g
S12 for crHCV-Tail 33%	TTCAGATCTGATTAAACAC (TS)<br>GTGTTTAATCAGATCTGAA (NTS)	Fig. 6e-g
S12 for crHCV-Tail 50%	TTCCGATCTGAGTAAACAC (TS)<br>GTGTTTACTCAGATCGGAA (NTS)	Fig. 6e-g
S12 AAAT PAM	TTAATGCTAACATTTGCG (TS)<br>CGCAAATGTTAGCATTAA (NTS)	Fig. 6b-d
S12 VVVN PAM	TTAATGCTAACNBBBGCG (TS)<br>CGCVVVNGTTAGCATTAA (NTS)	Fig. 6b-d

Table S4: List of protein sequences used in this study:

**Name**	**Sequence**
LbCas12a	MSKLEKFTNCYSLSKTLRFKAIPVGKTQENIDNKRLLVEDEKRAEDYKGVKKLLDRY<br>YLSFINDVLHSIKLKNLNNYISLFRKKTRTEKENKELENLEINLRKEIAKAFKGNEGYK<br>SLFKKDIIETILPEFLDDKDEIALVNSFNGFTTAFTGFFDNRENMFSEEAKSTSIAFRCIN<br>ENLTRYISNMDIFEKVDAIFDKHEVQEIKEKILNSDYDVEDFFEGEFFNFVLTQEGIDV<br>YNAIIGGFVTESGEKIKGLNEYINLYNQKTKQKLPKFKPLYKQVLSDRESLSFYGEGYT<br>SDEEVLEVFRNTLNKNSEIFSSIKKLEKLFKNFDEYSSAGIFVKNGPAISTISKDIFGEWN<br>VIRDKWNAEYDDIHLKKKAVVTEKYEDDRRKSFKKIGSFSLEQLQEYADADLSVVEK<br>LKEIIIQKVDEIYKVYGSSEKLFDADFVLEKSLKKNDAVVAIMKDLLDSVKSFENYIKA<br>FFGEGKETNRDESFYGDFVLAYDILLKVDHIYDAIRNYVTQKPYSKDKFKLYFQNPQF<br>MGGWDKDKETDYRATILRYGSKYYLAIMDKKYAKCLQKIDKDDVNGNYEKINYKL<br>LPGPNKMLPKVFFSKKWMAYYNPSEDIQKIYKNGTFKKGDMFNLNDCHKLIDFFKDS<br>ISRYPKWSNAYDFNFSETEKYKDIAGFYREVEEQGYKVSFESASKKEVDKLVEEGKL<br>YMFQIYNKDFSDKSHGTPNLHTMYFKLLFDENNHGQIRLSGGAELFMRRASLKKEEL<br>VVHPANSPIANKNPDNPKKTTTLSYDVYKDKRFSEDQYELHIPIAINKCPKNIFKINTE<br>VRVLLKHDDNPYVIGIDRGERNLLYIVVVDGKGNIVEQYSLNEIINNFNGIRIKTDYHS<br>LLDKKEKERFEARQNWTSIENIKELKAGYISQVVHKICELVEKYDAVIALEDLNSGFK<br>NSRVKVEKQVYQKFEKMLIDKLNYMVDKKSNPCATGGALKGYQITNKFESFKSMST<br>QNGFIFYIPAWLTSKIDPSTGFVNLLKTKYTSIADSKKFISSFDRIMYVPEEDLFEFALD<br>YKNFSRTDADYIKKWKLYSYGNRIRIFRNPKKNNVFDWEEVCLTSAYKELFNKYGIN<br>YQQGDIRALLCEQSDKAFYSSFMALMSLMLQMRNSITGRTDVDFLISPVKNSDGIFYD<br>SRNYEAQENAILPKNADANGAYNIARKVLWAIGQFKKAEDEKLDKVKIAISNKEWLE<br>YAQTSVKH
AsCas12a	MTQFEGFTNLYQVSKTLRFELIPQGKTLKHIQEQGFIEEDKARNDHYKELKPIIDRIYK<br>TYADQCLQLVQLDWENLSAAIDSYRKEKTEETRNALIEEQATYRNAIHDYFIGRTDNL<br>TDAINKRHAEIYKGLFKAELFNGKVLKQLGTVTTTEHENALLRSFDKFTTYFSGFYEN<br>RKNVFSAEDISTAIPHRIVQDNFPKFKENCHIFTRLITAVPSLREHFENVKKAIGIFVSTSI<br>EEVFSFPFYNQLLTQTQIDLYNQLLGGISREAGTEKIKGLNEVLNLAIQKNDETAHIIAS<br>LPHRFIPLFKQILSDRNTLSFILEEFKSDEEVIQSFCKYKTLLRNENVLETAEALFNELNS<br>IDLTHIFISHKKLETISSALCDHWDTLRNALYERRISELTGKITKSAKEKVQRSLKHEDI<br>NLQEIISAAGKELSEAFKQKTSEILSHAHAALDQPLPTTLKKQEEKEILKSQLDSLLGLY<br>HLLDWFAVDESNEVDPEFSARLTGIKLEMEPSLSFYNKARNYATKKPYSVEKFKLNF<br>QMPTLASGWDVNKEKNNGAILFVKNGLYYLGIMPKQKGRYKALSFEPTEKTSEGFD<br>KMYYDYFPDAAKMIPKCSTQLKAVTAHFQTHTTPILLSNNFIEPLEITKEIYDLNNPEK<br>EPKKFQTAYAKKTGDQKGYREALCKWIDFTRDFLSKYTKTTSIDLSSLRPSSQYKDLG<br>EYYAELNPLLYHISFQRIAEKEIMDAVETGKLYLFQIYNKDFAKGHHGKPNLHTLYW<br>TGLFSPENLAKTSIKLNGQAELFYRPKSRMKRMAHRLGEKMLNKKLKDQKTPIPDTL<br>YQELYDYVNHRLSHDLSDEARALLPNVITKEVSHEIIKDRRFTSDKFFFHVPITLNYQA<br>ANSPSKFNQRVNAYLKEHPETPIIGIDRGERNLIYITVIDSTGKILEQRSLNTIQQFDYQK

	KLDNREKERVAARQAWSVVGTIKDLKQGYLSQVIHEIVDLMIHYQAVVVLENLNFGF<br>KSKRTGIAEKAVYQQFEKMLIDKLNCLVLKDYPAEKVGGVLNPYQLTDQFTSFAKM<br>GTQSGFLFYVPAPYTSKIDPLTGFVDPFVWKTIKNHESRKHFLEGFDFLHYDVKTGDFI<br>LHFKMNRNLSFQRGLPGFMPAWDIVFEKNETQFDAKGTPFIAGKRIVPVIENHRFTGR<br>YRDLYPANELIALLEEKGIVFRDGSNILPKLLENDDSHAIDTMVALIRSVLQMRNSNA<br>ATGEDYINSPVRDLNGVCFDSRFQNPEWPMDADANGAYHIALKGQLLLNHLKESKD<br>LKLQNGISNQDWLAYIQELRN
ErCas12a	NNGTNNFQNFIGISSLQKTLRNALIPTETTQQFIVKNGIIKEDELRGENRQILKDIMDDY<br>YRGFISETLSSIDDIDWTSLFEKMEIQLKNGDNKDTLIKEQTEYRKAIHKKFANDDRFK<br>NMFSAKLISDILPEFVIHNNNYSASEKEEKTQVIKLFSRFATSFKDYFKNRANCFSADDI<br>SSSSCHRIVNDNAEIFFSNALVYRRIVKSLSNDDINKISGDMKDSLKEMSLEEIYSYEKY<br>GEFITQEGISFYNDICGKVNSFMNLYCQKNKENKNLYKLQKLHKQILCIADTSYEVPY<br>KFESDEEVYQSVNGFLDNISSKHIVERLRKIGDNYNGYNLDKIYIVSKFYESVSQKTYR<br>DWETINTALEIHYNNILPGNGKSKADKVKKAVKNDLQKSITEINELVSNYKLCSDDNI<br>KAETYIHEISHILNNFEAQELKYNPEIHLVESELKASELKNVLDVIMNAFHWCSVFMTE<br>ELVDKDNNFYAELEEIYDEIYPVISLYNLVRNYVTQKPYSTKKIKLNFGIPTLADGWSK<br>SKEYSNNAIILMRDNLYYLGIFNAKNKPDKKIIEGNTSENKGDYKKMIYNLLPGPNKM<br>IPKVFLSSKTGVETYKPSAYILEGYKQNKHIKSSKDFDITFCHDLIDYFKNCIAIHPEWK<br>NFGFDFSDTSTYEDISGFYREVELQGYKIDWTYISEKDIDLLQEKGQLYLFQIYNKDFS<br>KKSTGNDNLHTMYLKNLFSEENLKDIVLKLNGEAEIFFRKSSIKNPIIHKKGSILVNRT<br>YEAEEKDQFGNIQIVRKNIPENIYQELYKYFNDKSDKELSDEAAKLKNVVGHHEAAT<br>NIVKDYRYTYDKYFLHMPITINFKANKTGFINDRILQYIAKEKDLHVIGIDRGERNLIY<br>VSVIDTCGNIVEQKSFNIVNGYDYQIKLKQREGARQIARKEWKEIGKIKEIKEGYLSLV<br>IHEISKMVIKYNAIIVMEDLSYGFKKGRFKVERQVYQKFETMLINKLNYLVFKDISITE<br>NGGLLKGYQLTYIPDKLKNVGHQCGCIFYVPAAYTSKIDPTTGFVNIFKFKDLTVDAK<br>REFIKKFDSIRYDSEKNLFCFTFDYNNFITQNTVMSKSSWSVYTYGVRIKRRFVNGRFS<br>NESDTIDITKDMEKTLEMTDINWRDGHDLRQDIIDYEIVQHIFEIFRLTVQMRNSLSEL<br>EDRDYDRLISPVLNENNIFYDSAKAGDALPKDADANGAYCIALKGLYEIKQITENWKE<br>DGKFSRDKLKISNKDWFDFIQNKRYL

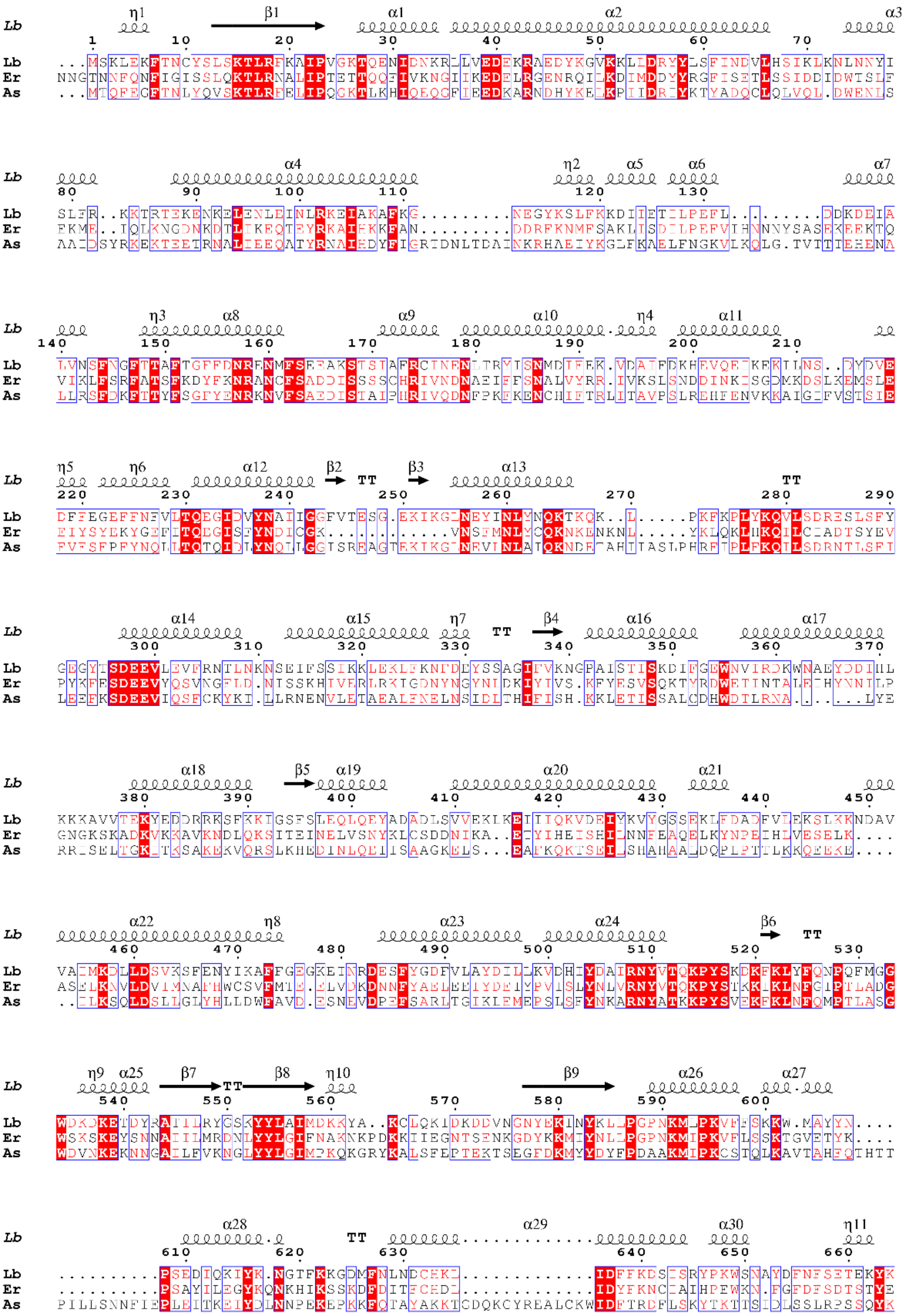

Lb
Er
As

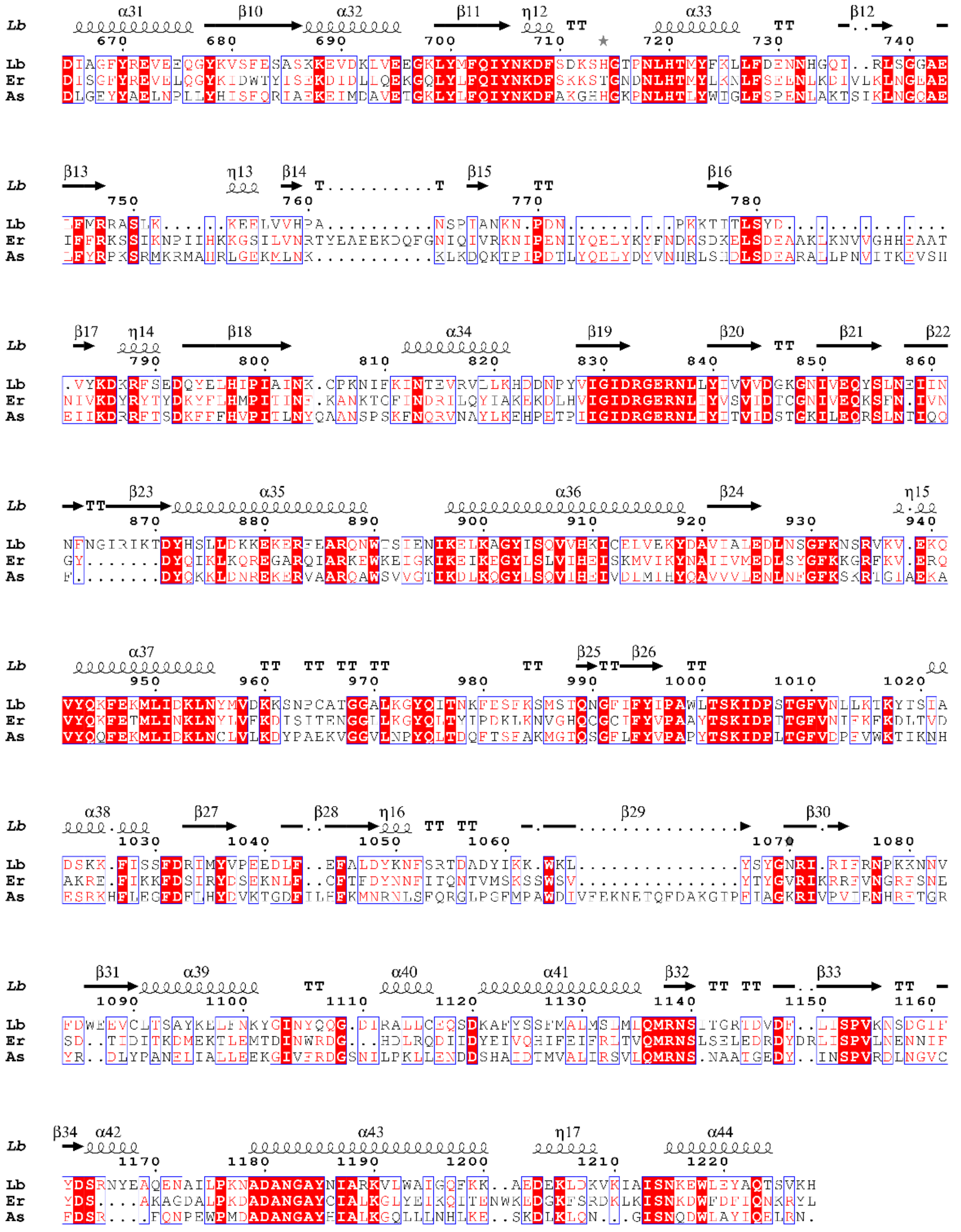

Fig. S1: Multiple sequence alignment of Lb, As, and Er Cas12a orthologs are shown. The alignment was performed using MultiAlin. The alignment file was imported into Esprit 3.0[1] and aligned against the LbCas12a structure (PDB: 5XUS)[2].

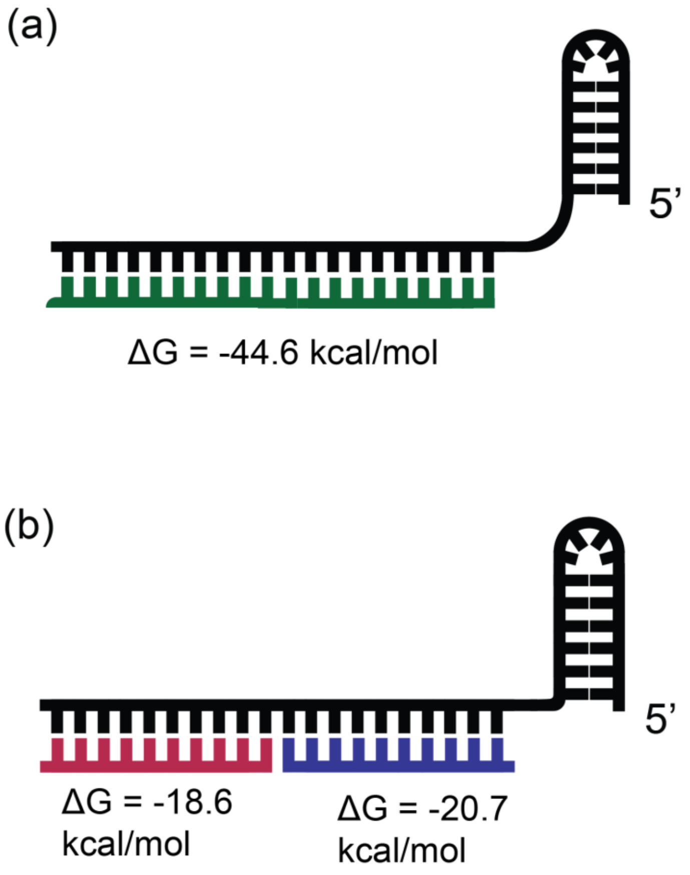

Fig.S2: Computationally predicted Gibbs free energy change for the binding of **(a)** a full-length 20-nt activator to the crRNA and **(b)** two short activators of length 10-nt each binding to different regions of the crRNA in a 'split-activator' fashion. Predictions were made using DINAMELT[3].

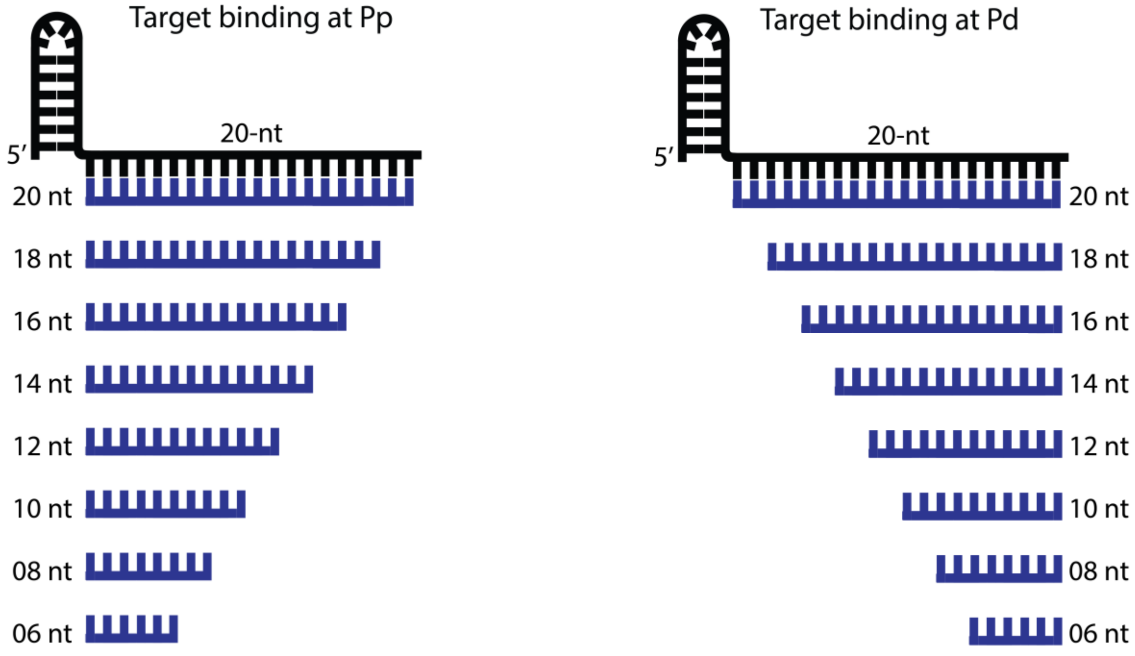

Fig. S3: Schematic depicting how truncated ssDNA activators of different length bind to the PAM proximal (Pp) and PAM distal regions of the crRNA.

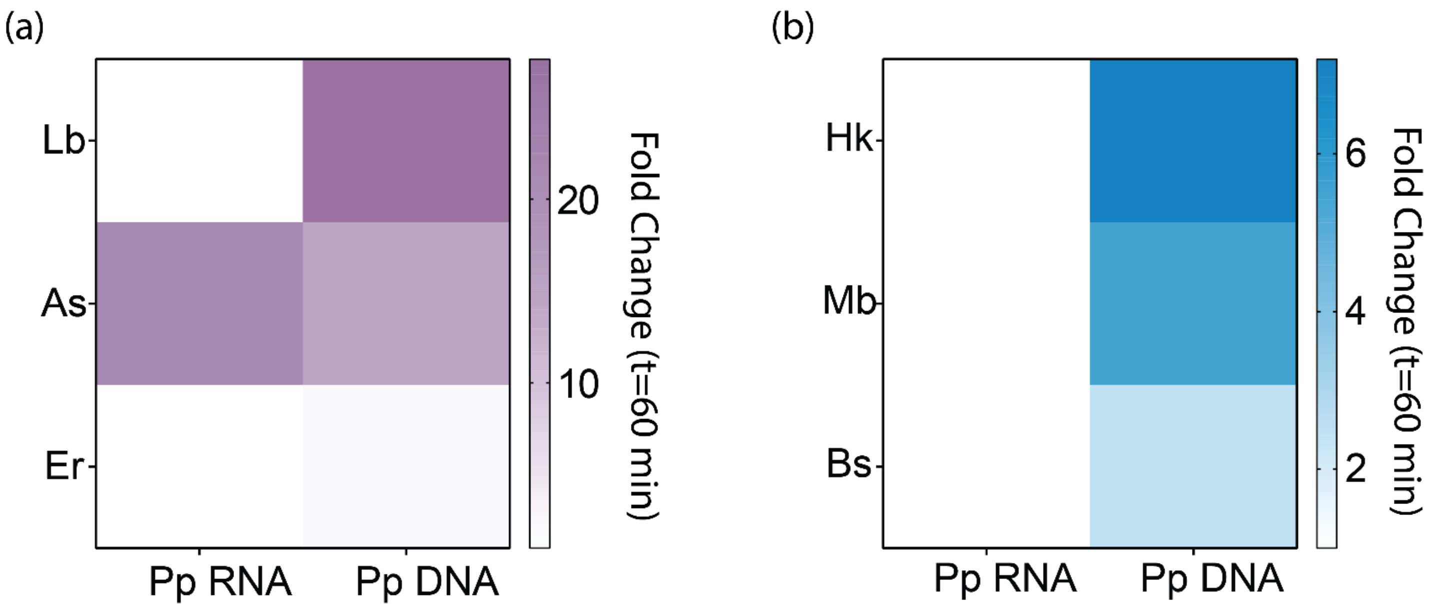

Fig.S4: Heat map representing the fold change in fluorescence with respect to the NTC for a split-activator combination of 10-nt PAM-distal (Pd) DNA and either a 10-nt PAM-proximal (Pp) RNA or an equivalent Pp DNA. The experiments were performed with 6 different orthologs of Cas12a – Lb, As, Er (left) and Hk, Mb, Bs (right). Source data are provided as a Source Data file.

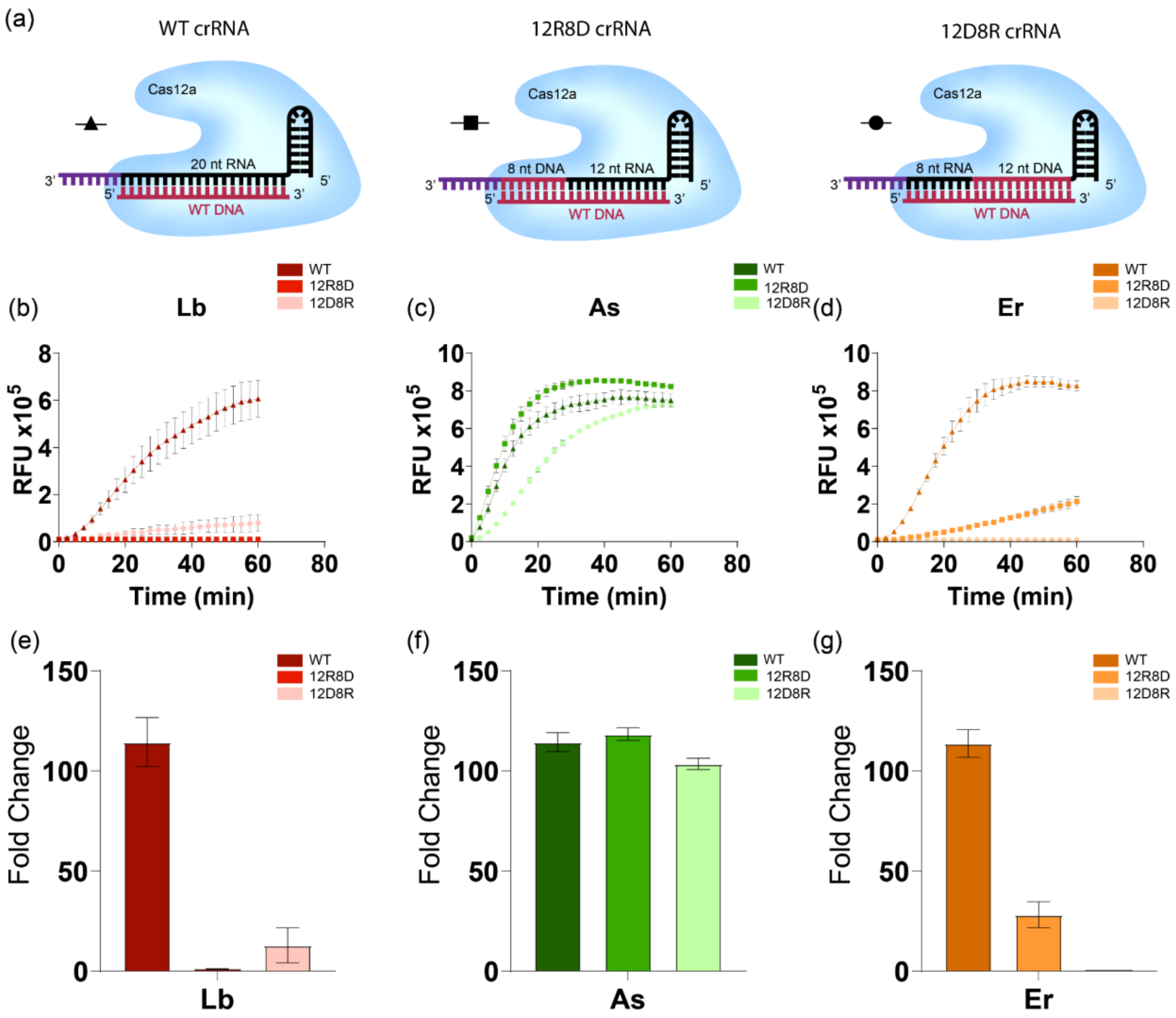

Fig S5: Chimeric DNA-RNA guides complexed with Cas12a. (a) Schematic representation of chimeric DNA-RNA hybrid crRNAs complexed with Cas12a and activated with WT ssDNA activators. Chimeric crRNA was designed by changing 12-nt near the PAM-proximal 5'-end of the crRNA to DNA (12D8R crRNA) and changing the PAM distal 8-nt end of the crRNA to DNA (12R8D crRNA). WT crRNA is represented in graphs WT crRNA is represented on graphs b-d by triangles, 12R8D crRNA is represented by squares, and 12D8R crRNA is represented by circles. (b-d) Relative RFU values of in vitro *trans*-cleavage assay with Cas12a orthologs (Lb- red, As- green, Er- orange) complexed with WT crRNA, 12D8R crRNA, and 12R8D crRNAs. (e-g) Fold change at 60 min is represented for each crRNA and three Cas proteins. The reactions contained 25 nM ssDNA GFP WT activator, 60 nM Cas12a, and 12 nM crRNA (WT, 12R8D, 12D8R). Reactions were incubated for 60 min at 37°C. Error bars represent mean value +/- SD (n=3). Source data are provided as a Source Data file.

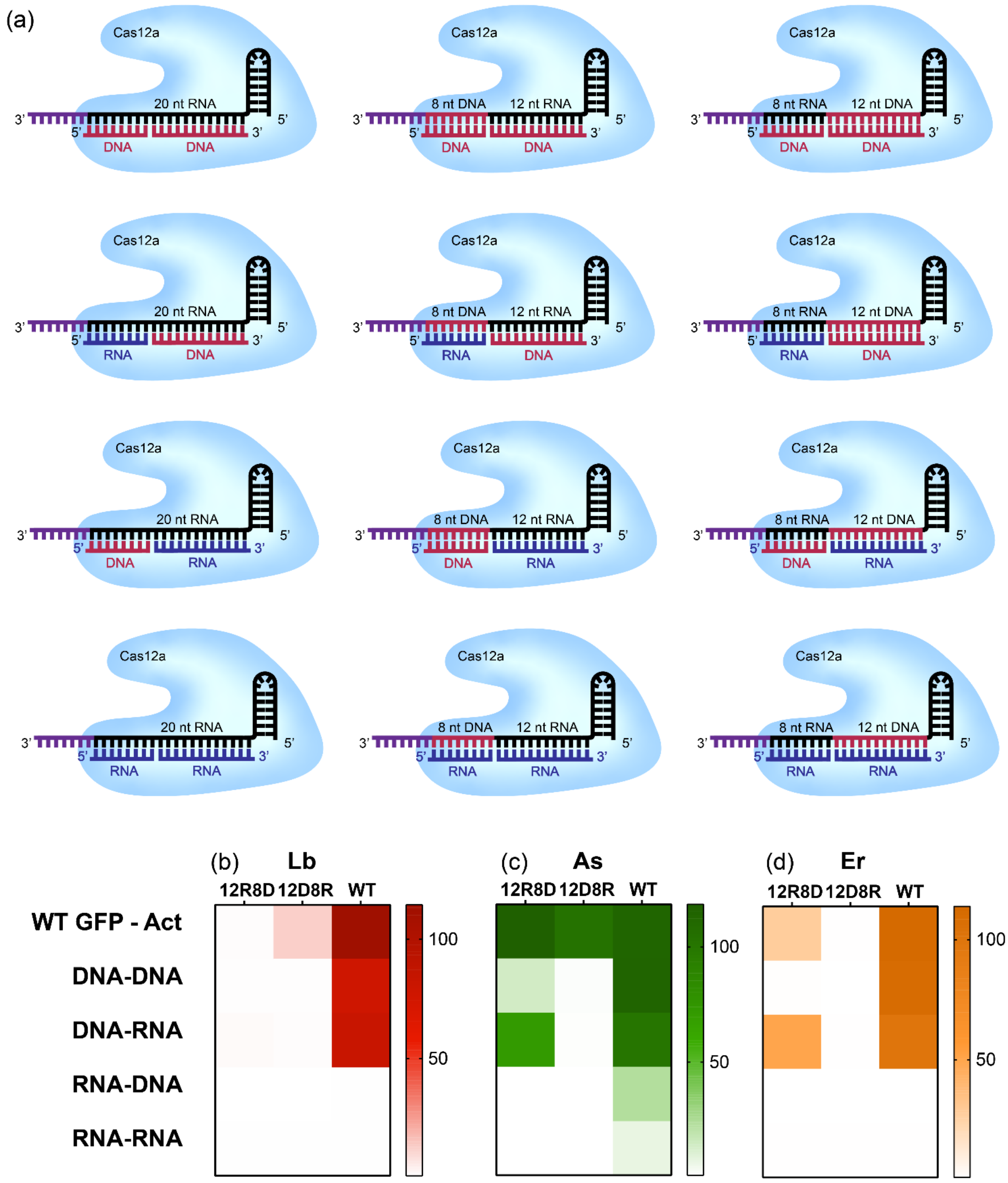

Fig S6: Reverse transcription-free RNA detection with Cas12a and ‘split activator’ mechanism. (a) Schematic representation of Cas12a complexed with chimeric crRNAs and activated by ‘split activator’ system. Chimeric crRNAs include 12D8R and 12R8D crRNAs as well as WT crRNA. Combinations of activators include ssDNA and RNA targeting the PAM proximal and distal locations on the crRNA. (b-d) Heat maps representing the fold changes of in vitro *trans*-cleavage assay with Cas12a orthologs complexed with WT and chimeric crRNAs. Combinatorial schemes for the ‘split activator system’ are seen in (a) (n=3). Source data are provided as a Source Data file.

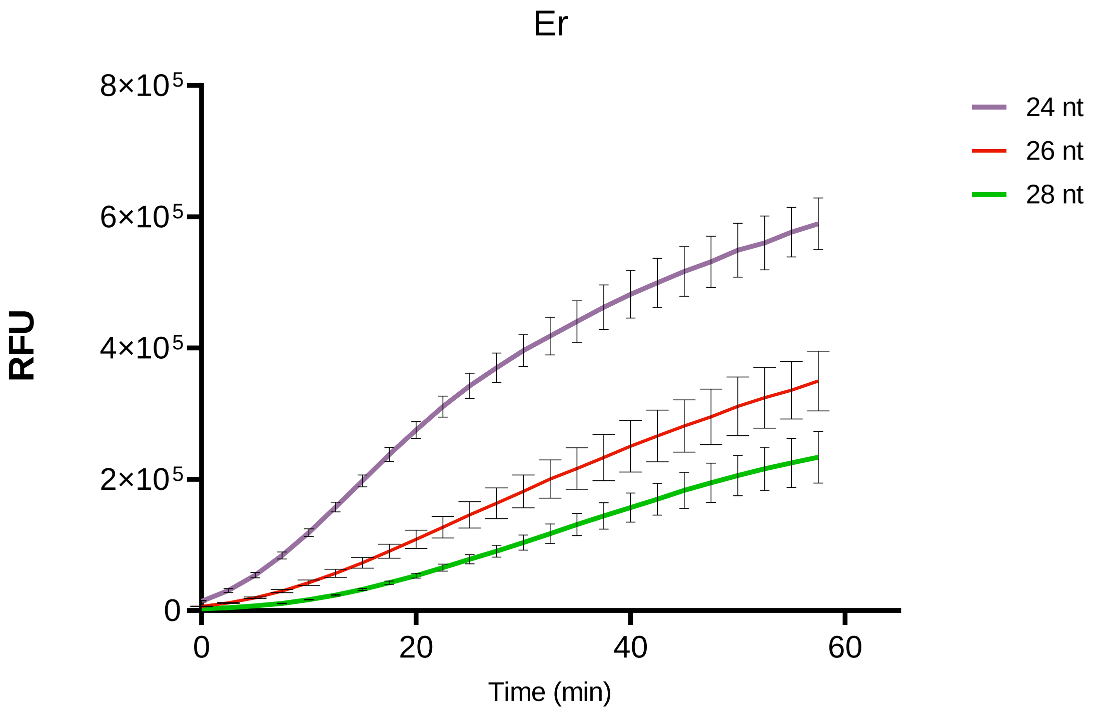

Fig S7: Increasing lengths of the crRNA ranging from 24-26 nt were tested for HCV RNA detection with ErCas12a based SAHARA. For each length of the crRNA, the S12 activator was kept at a constant length of 12-nt while the target HCV RNA was varied from 12-16 nt to enable increasing amount of target binding to the crRNA. The crRNA of length 24-nt that bound to 12-nt of S12 DNA and 12-nt of RNA target showed the highest activity. Error bars represent mean value +/- SD (n=3). Source data are provided as a Source Data file.

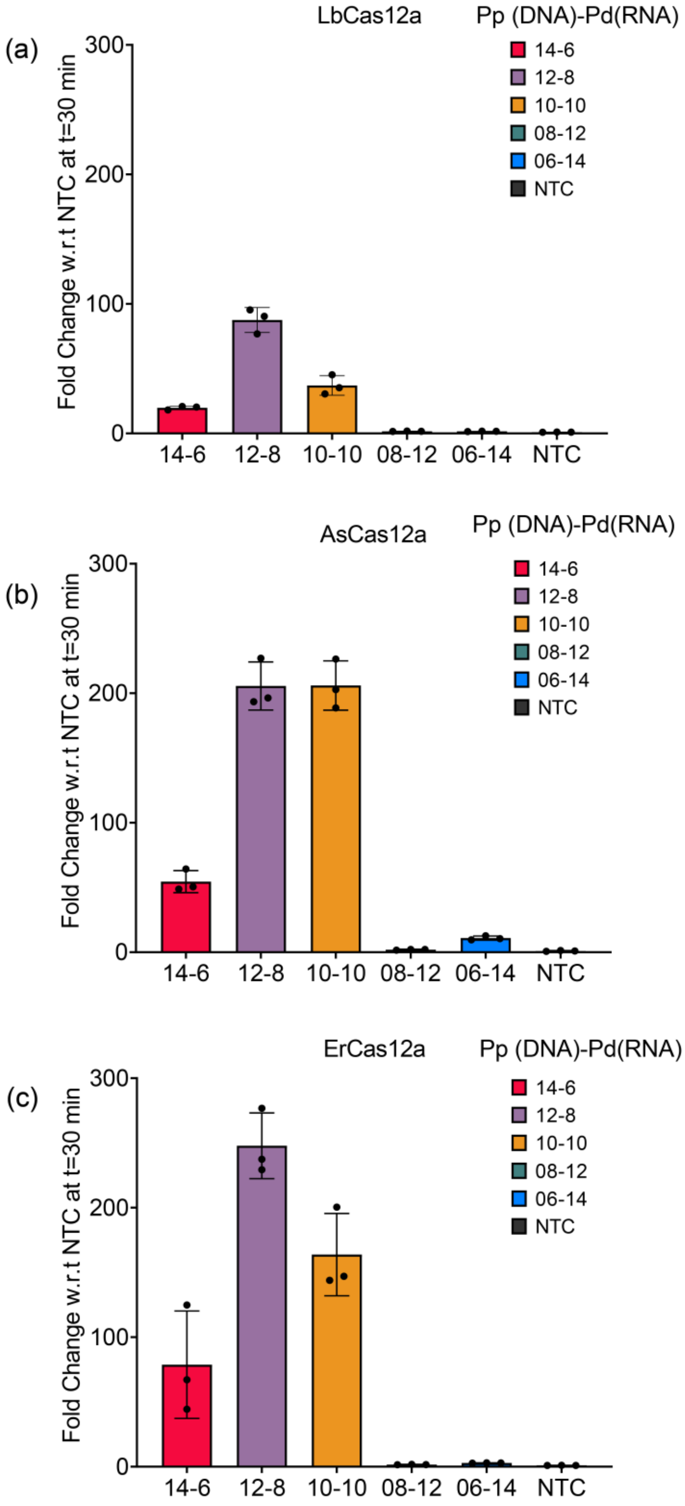

Fig S8: For a 20-nt crRNA, the length of the Pp binding DNA activator and the Pd binding RNA activator was varied from 6-14 nt. RNA detection was only tolerated for RNA activators of length 6nt-8nt, but not for RNA of length 12-nt or 14-nt. Error bars represent mean value +/- SD (n=3). Source data are provided as a Source Data file.

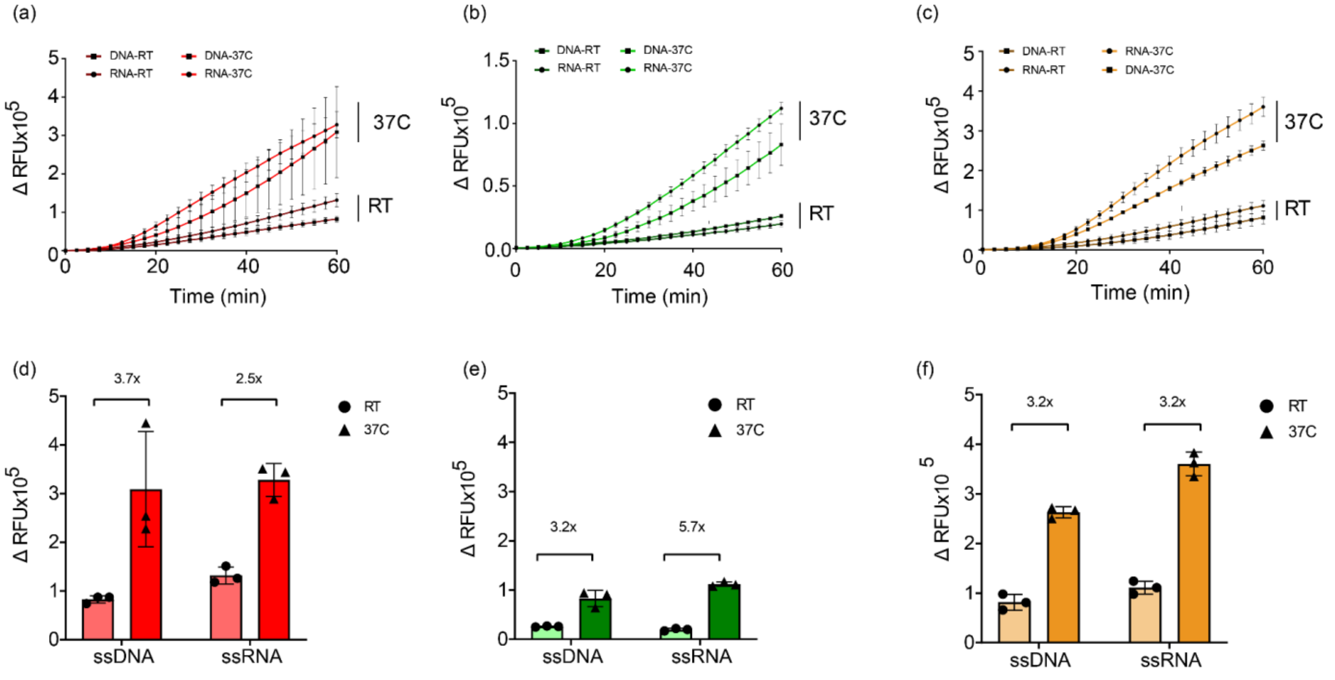

Fig S9: Detection of ssDNA or ssRNA sequences with SAHARA at different temperatures. **a-c:** Raw fluorescence data showing the *trans*-cleavage activity of SAHARA for the detection of an ssDNA or ssRNA sequence at either room temperature (RT) or 37°C for Lb (red), As (green), and Er (orange) orthologs. Error bars represent S.D. (n=3). **d-f:** Background subtracted raw fluorescence intensity for the detection of ssDNA or ssRNA sequences at RT or 37°C. Error bars represent S.D. (n=3). Error bars for all charts represent mean value +/- SD (n=3). Source data are provided as a Source Data file.

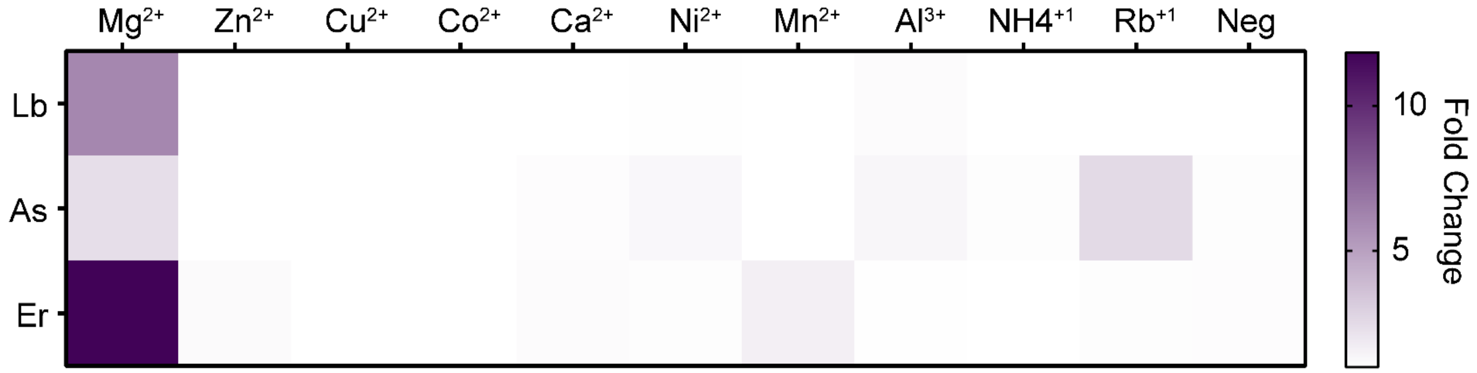

Fig S10: Optimization of SAHARA with different divalent metal ions (a) Effect of different metal ions on the SAHARA with Lb, As, and Er Cas12a enzymes. Negative control represents a no-salt buffer. Each metal ion buffer contains 3 mM of the respective metal salt. The heat map indicates fold change at time t=60 min (n=3). Source data are provided as a Source Data file.

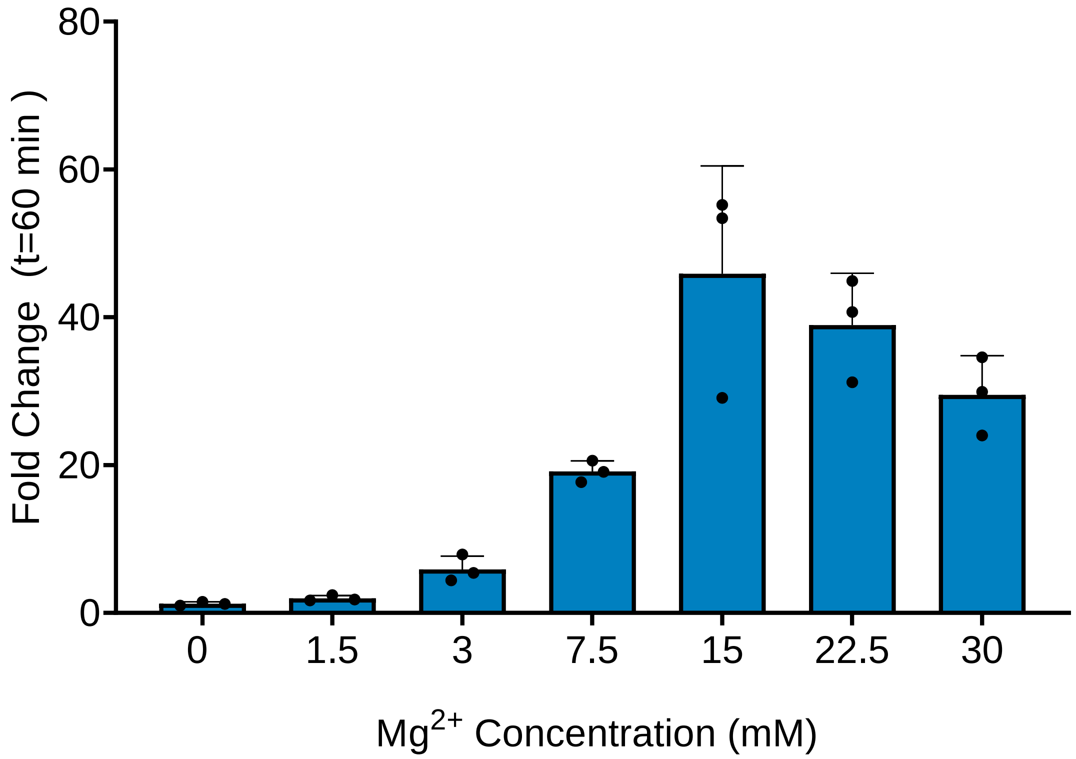

Fig S11: Optimization of SAHARA with Mg ion concentration: The *trans*-cleavage activity of SAHARA with ErCas12a under increasing Mg^{2+} ion concentration is shown. The plot of fold change in RFU compared to NTC at t=60 min is shown. Error bars represent mean value +/- SD (n=3). Source data are provided as a Source Data file.

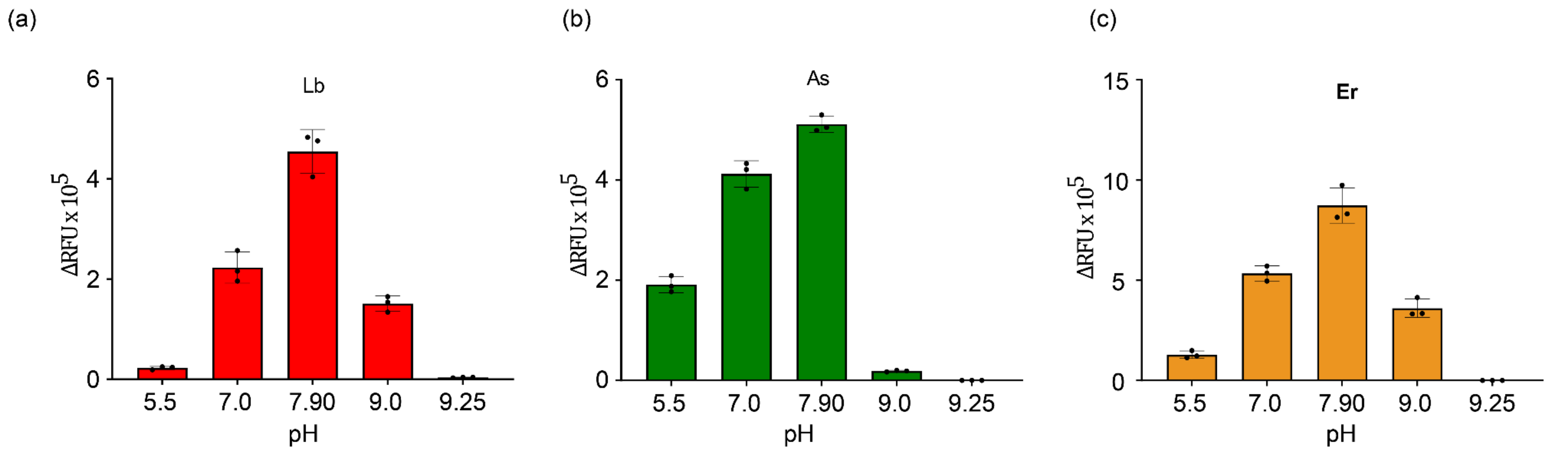

Fig S12. Optimization of SAHARA with pH conditions a-c: Effect of buffer pH on the *trans*-cleavage activity of SAHARA is shown. Bar graphs indicate background subtracted RFU at time t=60 min for Lb, As, and Er Cas12a orthologs at a different range of pH values. Error bars represent mean value +/- SD (n=3). Source data are provided as a Source Data file.

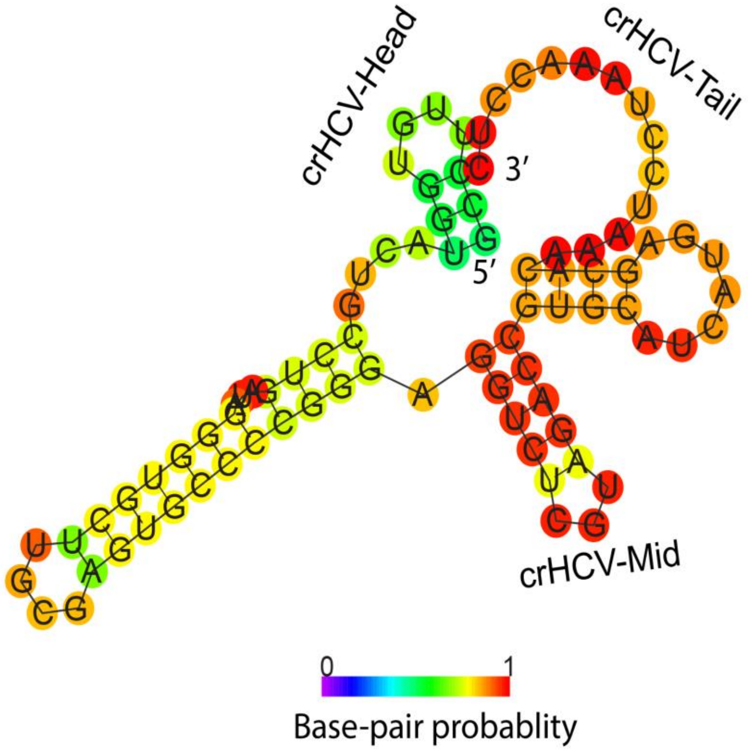

Fig S13. Computationally predicted secondary structure of the HCV polypeptide precursor targets. The three regions being targeted by SAHARA are labeled as HCV-Head (GCCUUGUGGUAC), HCV-Mid (AGACCGUGCAUC), and HCV-Tail (AUCCUAAACCUC) respectively. Predictions were done using the RNAFold webserver.

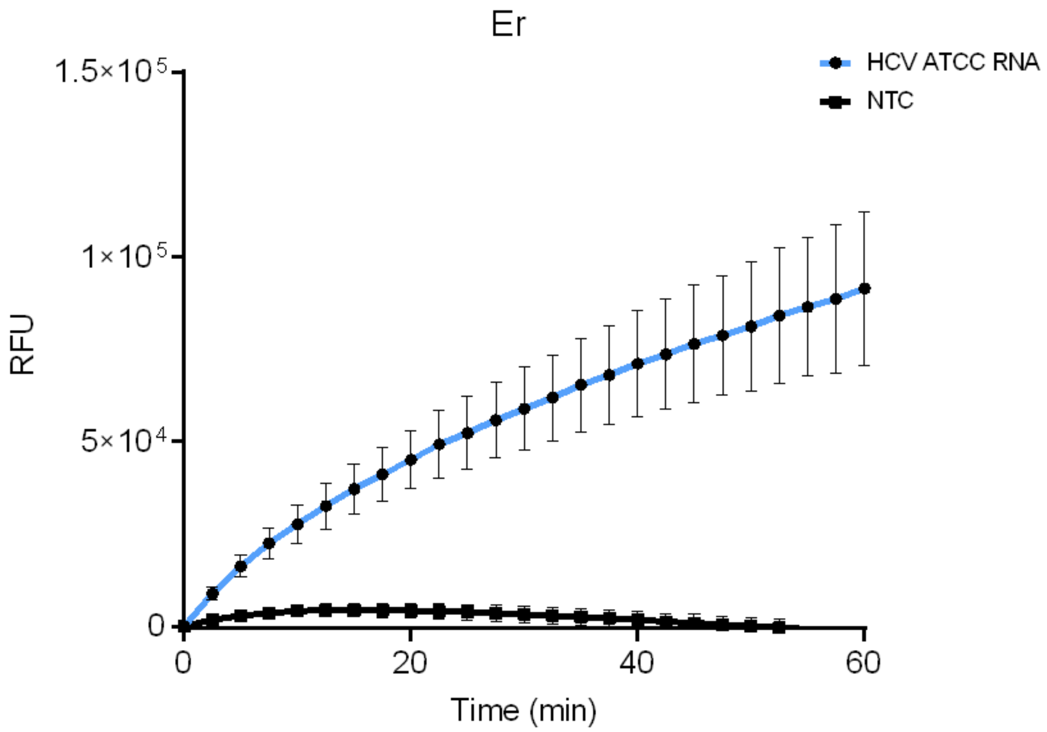

Fig S14: Quantitative Synthetic RNA from Hepatitis C Virus purchased from ATCC (HCV-ATCC) was tested for detection with ErCas12a based SAHARA. 250 nM of a crRNA pool consisting of HCV-Tail, HCV-Mid and HCV-Head; 125 nM Cas12a; 500 nM FQ and 105 nM S12-DNA was used in the reaction. Data represents an increase in RFU vs time for a time-course of 1 hr for HCV-ATCC target and a No Target Control (NTC). Error bars represent mean value +/- SD (n=3). Source data are provided as a Source Data file.

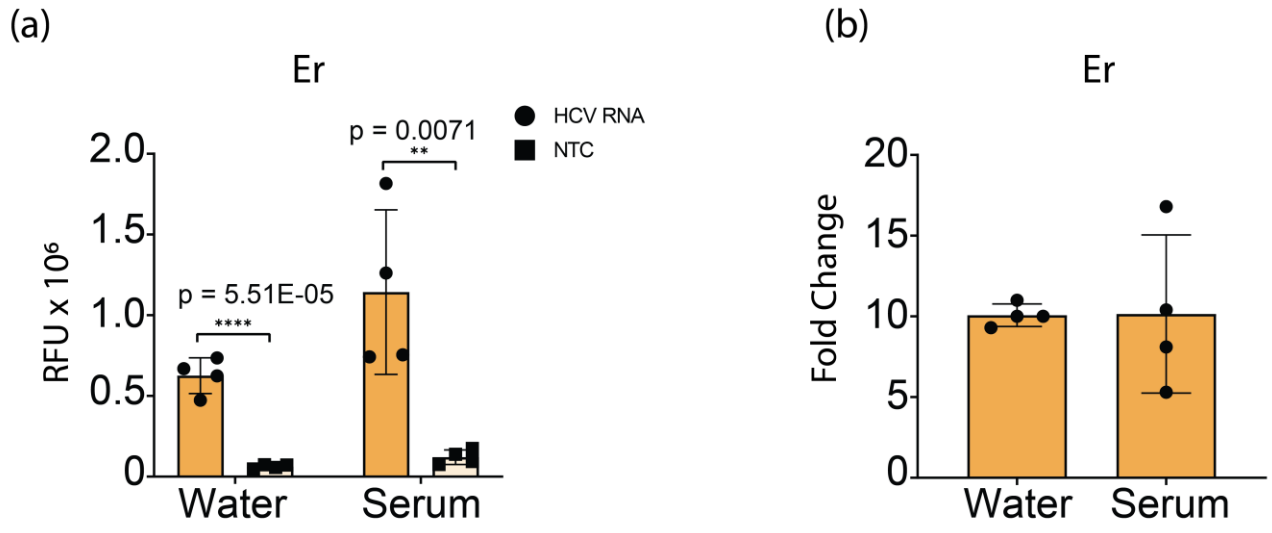

Fig S15: Target HCV RNA was spiked in a matrix consisting of either water or nucleic acid extract from healthy serum sample to a final concentration of 1 nM. The detection efficiency of target RNA spiked in water or serum was determined with ErCas12a based SAHARA. (a) Plot representing RFU of target RNA detection in water or serum at t=60 min. Error bars represent S.D. (n=4) (b) Plot representing fold change of fluorescence intensity from target RNA sample with respect to the fluorescence from the no target control (NTC) in water and serum. Error bars represent mean value +/- SD (n=4). Statistical analysis for n=4 biologically independent replicates was performed using a two-tailed t-test where ns = not significant with $p > 0.05$, and the asterisks (* $P \leq 0.05$, ** $P \leq 0.01$,*** $P \leq 0.001$, **** $P \leq 0.0001$) denote significant differences. Source data are provided as a Source Data file.

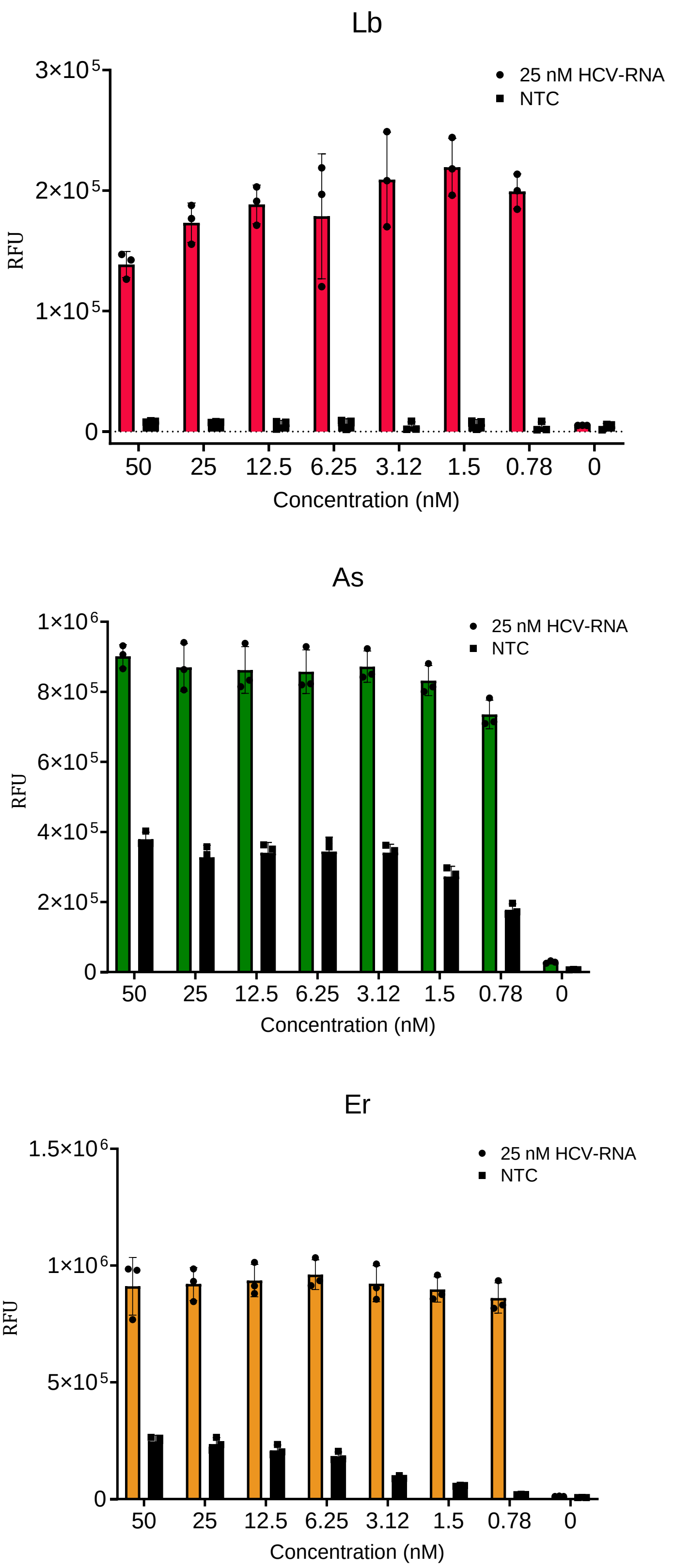

Fig. S16: Effect of S12 concentration ranging from 50 nM -780pM on the *trans*-cleavage activity of SAHARA is shown for Lb, As, and Er Cas12a orthologs. The plot of RFU at t=60 min in the presence or absence of 25 nM target HCV-RNA and different S12 concentrations is

shown. Error bars represent mean value +/- SD (n=3). Source data are provided as a Source Data file.

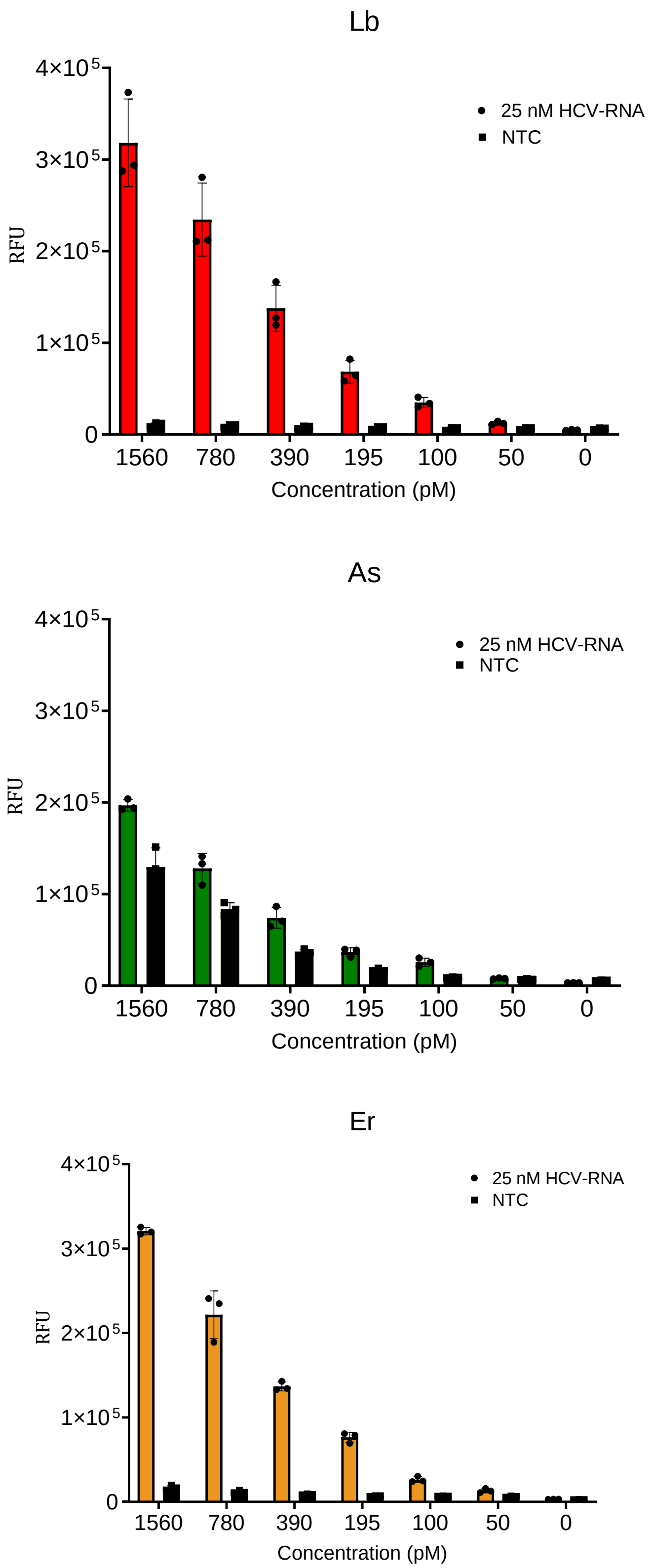

Fig. S17: Effect of S12 concentration ranging from 1.56 nM to 50 pM on the *trans*-cleavage activity of SAHARA is shown for Lb, As, and Er Cas12a orthologs. A plot of RFU at t=60 min in the presence or absence of 25 nM target HCV-RNA and different S12 concentrations is shown. Error bars represent mean value +/- SD (n=3). Source data are provided as a Source Data file.

References

1. Robert, X. & Gouet, P. Deciphering key features in protein structures with the new ENDscript server. *Nucleic Acids Res.* **42**, W320–W324 (2014).
2. Yamano, T. *et al.* Structural Basis for the Canonical and Non-canonical PAM Recognition by CRISPR-Cpf1. *Mol. Cell* **67**, 633-645.e3 (2017).
3. Markham, N. R. & Zuker, M. DINAMelt web server for nucleic acid melting prediction. *Nucleic Acids Res.* **33**, W577–W581 (2005).
