# Supplementary material for: Programmable RNA detection with CRISPR-Cas12a
Source: Nat Commun. 2023 Sep 5;14:5409. doi: 10.1038/s41467-023-41006-1 (PMC10480431; doi:10.1038/s41467-023-41006-1)
Supplement: Supplementary file 2 — Reporting Summary [file 41467_2023_41006_MOESM2_ESM.pdf]

nature portfolio

Corresponding author(s): Dr. Piyush K. Jain

Last updated by author(s): Aug 14, 2023

Reporting Summary

Nature Portfolio wishes to improve the reproducibility of the work that we publish. This form provides structure for consistency and transparency in reporting. For further information on Nature Portfolio policies, see our Editorial Policies and the Editorial Policy Checklist.

Statistics

For all statistical analyses, confirm that the following items are present in the figure legend, table legend, main text, or Methods section.

n/a	Confirmed	
☐	☒	The exact sample size (*n*) for each experimental group/condition, given as a discrete number and unit of measurement
☐	☒	A statement on whether measurements were taken from distinct samples or whether the same sample was measured repeatedly
☐	☒	The statistical test(s) used AND whether they are one- or two-sided<br>*Only common tests should be described solely by name; describe more complex techniques in the Methods section.*
☒	☐	A description of all covariates tested
☒	☐	A description of any assumptions or corrections, such as tests of normality and adjustment for multiple comparisons
☐	☒	A full description of the statistical parameters including central tendency (e.g. means) or other basic estimates (e.g. regression coefficient) AND variation (e.g. standard deviation) or associated estimates of uncertainty (e.g. confidence intervals)
☐	☒	For null hypothesis testing, the test statistic (e.g. *F*, *t*, *r*) with confidence intervals, effect sizes, degrees of freedom and *P* value noted<br>*Give P values as exact values whenever suitable.*
☒	☐	For Bayesian analysis, information on the choice of priors and Markov chain Monte Carlo settings
☒	☐	For hierarchical and complex designs, identification of the appropriate level for tests and full reporting of outcomes
☒	☐	Estimates of effect sizes (e.g. Cohen's *d*, Pearson's *r*), indicating how they were calculated

Our web collection on statistics for biologists contains articles on many of the points above.

Software and code

Policy information about availability of computer code

Data collection	No specific software used to collect the data.
Data analysis	Only publicly/commercially available softwares used<br>General: Graphpad Prism version 9.5.0 (730) was used to analyze data and plot figures.<br>Figure S1: Multiple sequence alignments was performed using MultiAlin. The alignment file was imported into Esprit 3.048 software and aligned against the LbCas12a structure (PDB ID: 5XUS). RCSB PDB was used to obtain the structural information of Cas enzymes.<br>Figure S2: Gibbs free energy change predictions were made using DINAMELT in Figure S2.<br>Figure S12: RNA folding structure predictions were performed using the RNAFold webserver.

For manuscripts utilizing custom algorithms or software that are central to the research but not yet described in published literature, software must be made available to editors and reviewers. We strongly encourage code deposition in a community repository (e.g. GitHub). See the Nature Portfolio guidelines for submitting code & software for further information.

Data

Policy information about availability of data

All manuscripts must include a data availability statement. This statement should provide the following information, where applicable:

- Accession codes, unique identifiers, or web links for publicly available datasets
- A description of any restrictions on data availability
- For clinical datasets or third party data, please ensure that the statement adheres to our policy

All the data supporting the findings of this study are available within the Article and Supplementary Files. Source data is available in the Source Data file. The crystal structure of LbCas12a enzyme (PDB ID: 5XUS) deposited by Yamano et. al. can be obtained from Protein Data Bank (PDB) by following this link: https://www.rcsb.org/structure/5xus

Human research participants

Policy information about studies involving human research participants and Sex and Gender in Research.

Reporting on sex and gender	N/A
Population characteristics	N/A
Recruitment	N/A
Ethics oversight	N/A

Note that full information on the approval of the study protocol must also be provided in the manuscript.

Field-specific reporting

Please select the one below that is the best fit for your research. If you are not sure, read the appropriate sections before making your selection.

☒ Life sciences ☐ Behavioural & social sciences ☐ Ecological, evolutionary & environmental sciences

For a reference copy of the document with all sections, see nature.com/documents/nr-reporting-summary-flat.pdf

Life sciences study design

All studies must disclose on these points even when the disclosure is negative.

Sample size	n=3 replicates were used for all experiments (unless otherwise stated), as is standard for the field based on prior publications including Chen et al., Science, (2018). DOI:10.1126/science.aar6245
Data exclusions	No data were excluded.
Replication	All experiments were performed in triplicates. All replications were successful.
Randomization	Randomization does not apply to this study.
Blinding	Randomization does not apply to this study.

Reporting for specific materials, systems and methods

We require information from authors about some types of materials, experimental systems and methods used in many studies. Here, indicate whether each material, system or method listed is relevant to your study. If you are not sure if a list item applies to your research, read the appropriate section before selecting a response.

Materials & experimental systems

n/a	Involved in the study
☒	☐ Antibodies
☒	☐ Eukaryotic cell lines
☒	☐ Palaeontology and archaeology
☒	☐ Animals and other organisms
☒	☐ Clinical data
☒	☐ Dual use research of concern

Methods

n/a	Involved in the study
☒	☐ ChIP-seq
☒	☐ Flow cytometry
☒	☐ MRI-based neuroimaging
